# A single shot coherent Ising machine based on a network of injection-locked multicore fiber lasers

**DOI:** 10.1038/s41467-019-11548-4

**Published:** 2019-08-06

**Authors:** Masoud Babaeian, Dan T. Nguyen, Veysi Demir, Mehmetcan Akbulut, Pierre-A Blanche, Yushi Kaneda, Saikat Guha, Mark A. Neifeld, N. Peyghambarian

**Affiliations:** 1 College of Optical Sciences, University of Arizona, Tucson, AZ 85721 USA; 2 Department of Physics, University of Arizona, Tucson, AZ 85721 USA; 3 Corning Research and Development Corporation, Corning, NY 14831 USA; 4 Department of Electrical and Computer Engineering, University of Arizona, Tucson, AZ 85721 USA; 5 Present Address: ASML Corp, Wilton, CT 06897 USA

**Keywords:** Physics, Nonlinear optics, Fibre optics and optical communications

## Abstract

Combinatorial optimization problems over large and complex systems have many applications in social networks, image processing, artificial intelligence, computational biology and a variety of other areas. Finding the optimized solution for such problems in general are usually in non-deterministic polynomial time (NP)-hard complexity class. Some NP-hard problems can be easily mapped to minimizing an Ising energy function. Here, we present an analog all-optical implementation of a coherent Ising machine (CIM) based on a network of injection-locked multicore fiber (MCF) lasers. The Zeeman terms and the mutual couplings appearing in the Ising Hamiltonians are implemented using spatial light modulators (SLMs). As a proof-of-principle, we demonstrate the use of optics to solve several Ising Hamiltonians for up to thirteen nodes. Overall, the average accuracy of the CIM to find the ground state energy was ~90% for 120 trials. The fundamental bottlenecks for the scalability and programmability of the presented CIM are discussed as well.

## Abstract (web summary)

For specific computation problems where electronic digital processors have shortcomings in simulating, an analog optical system may be a solution, Here, the authors present an analog all-optical implementation of a coherent Ising machine based on a network of injection-locked multicore fiber lasers.

## Introduction

For decades, optics has been preferred for communication and parallel processing^1^. In the past, optical-computing techniques have been able to demonstrate some mathematical operations such as Fourier Transform^2^, vector matrix multiplication^3^, inverse matrix^4–6^, and more recently multiplication and division using nonlinear optics^7–10^. One of today’s major challenges in the digital electronic computation is the optimization problem for a very large data set. To improve the power consumption and speed for that type of computation, several new technologies have been introduced. For instance, multicore for electronic central processing units as well as parallel computing architecture such as subthreshold very large-scale integration (VLSI), application-specific integrated circuit (ASIC), and a custom ASIC, the Tensor Processing Unit (TPU)^11^. However, optimization problems for large data sets remain an issue and is a subject of ongoing research both in terms of software and hardware improvements. The main problems faced by electronic computation platforms are bandwidth limitation and high power consumption of electronic devices^1,12,13^. Hybrid optical-electronic platforms have been recently explored as a way to enhance the speed, and to lower the power consumption for some computation problems. Examples include reservoir computing^14–16^, signal processing^17–22^, and spike processing^23–25^.

Optical computers do not have to mimic the same algorithmic design used in digital computers. Early attempts to do so failed to implement an all optical computing based on optical logical gates due to the lack of energy-efficient and compact optical devices^26^. In some physical systems, remarkably in ultrashort laser phenomena, the nonlinear dynamics of a complex system rapidly happen much faster than what can be computed by a digital computer. For such problems, an analog device that mimics the physics of a complex phenomenon may have great benefits for computational purposes^17,18^. In other words, for specific computation problems where electronic digital processors have difficulties in simulating a complex nonlinear system, an analog optical system may be a solution, or can be used as an accelerator, to help the digital simulation in a metaphoric way via a non-algorithmic approach, where an engineered programmable all-optical computer serves as a metaphor to the desired nonlinear dynamics to be emulated^27–31^.

Combinatorial optimization problems are universal and have many applications from image processing^32^, artificial intelligence^33,34^, machine learning^34–37^ to computational biology^38–44^. Most of these problems are in non-deterministic polynomial time (NP)-hard or NP-complete categories. There exist some approximate algorithms^45–47^ or simulated annealing methods^48^ that are used commonly in digital computers to obtain a solution in reasonable time. However, solving these problems efficiently in terms of power consumption and speed for a large number of variables (e.g. 10^6^), is still beyond classical digital computers and electronic analog computation platforms alike^7–9^. Quantum annealing^49–51^ and adiabatic quantum computation platforms^52^ have been recently introduced in the effort of solving NP-hard problems. Although, the performance and scalability of such machines still need to be explored^53,54^. Finding the ground state spin configuration of the general Ising Hamiltonian is known to be an NP-hard problem (for three dimensions, as well as two dimensions with the Zeeman term):^55^

1 $$H = \mathop {\sum }\limits_{i,j \ne i}^N J_{ij}\sigma _i\sigma _j + \mathop {\sum }\limits_{i = 1}^N \lambda _i\sigma _i$$

Here, *H* is the Ising Hamiltonian, *J*_*ij*_ is the mutual couplings between node *i* and *j*, *λ*_*i*_ is called the Zeeman term (external field), *σ*_*i*_ and *σ*_*j*_ are the *i*th and the *j*th spins, respectively, where each spin can take a value of +1 or −1. In the past few years, some physical systems have been introduced as coherent Ising Hamiltonian solvers. One such system was based on a coupled degenerate optical parametric oscillator network^56–60^, and another was based on a network of injection-locked lasers^61–64^.

In this paper, we present simulations as well as experimental results for an all-optical analog coherent Ising machine (CIM) based on a network of injection-locked single frequency multicore fiber (MCF) lasers. As a proof-of-principle, we have performed the experiment for several Ising Hamiltonians with size of *N* = 3 (triangle topology), *N* = 4 (square lattice), *N* = 7 (1D chain), and *N* = 13 example. In the optical platform we present, three spatial light modulators (SLMs) are used, one to program the Zeeman terms, the two others to encode the mutual couplings. Our experimental results are compared with a brute-force algorithm (BFA) which performs an exhaustive search to find the exact ground state of the Ising Hamiltonian. Statistical analysis shows that our system achieved an average accuracy of ~90% for 120 trials. We examined four different Ising Hamiltonians and repeated the experiment up to 20 times for each case. The accuracy is calculated based on the following:

2 $${\mathrm{Accuracy}} = \left| {\frac{{E_{{\mathrm{max}}} - E_{{\mathrm{exp}}}}}{{E_{{\mathrm{max}}} - E_{\mathrm{G}}}}} \right| \times 100{\mathrm{\% }},$$

where *E*_exp_ is the measured expectation value of the Ising Hamiltonian based on the experiment. *E*_G_ and *E*_max_ are the calculated ground state and maximum expectation values of the Ising Hamiltonian, respectively, according to BFA simulation.

The modeling of the system has been done based on evolution of complex photon field operator and approximating the photon field amplitude by a square root of the photon number (mean field approximation) in right and left circular polarizations. Finally, we discuss some bottlenecks in the scalability, accuracy, and programmability of the presented CIM.

We would like to stress that the theoretical and experimental results in this paper exhibit an interesting candidate platform for an optical computer whose coupled nonlinear analog dynamics exhibits a discrete binary saturation effect that seeks a physical energy minimum^61^. This makes it a strong candidate to encode binary-valued Boolean optimization problems. However, whether the physical energy minimum maps to the computational energy minimum (e.g., of an Ising problem) producing results of sufficient accuracy that cannot be obtained using digital means, e.g., using a polynomial time approximation scheme (PTAS), remains unclear. However, the small-size systems we simulate and physically emulate show excellent agreement with theory, and produce results with remarkable accuracy to attaining the ground state of the Ising function, and consumes much less power than a purely digital solver would consume.

## Results

### Injection-locked laser system and mapping to the Ising Hamiltonian

**Fig. 1:**
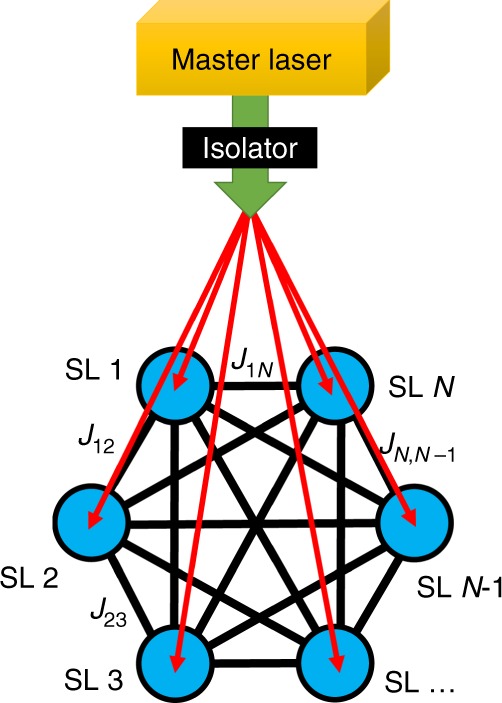
Injection-locked laser system scheme. *N* as the number of slave lasers (SLs) denotes the size of the Ising Hamiltonian and the optical interactions pathway between them follow the mutual couplings concept. Master laser (ML) initiates the Ising Hamiltonian with slightly change to its vertical linear polarization state

The system is composed of an injection-locked laser network where several slave lasers (SLs) are locked to a master laser (ML) in terms of oscillation frequency and polarization state. The number of SLs represent the number of nodes in the Ising Hamiltonian, and they are locked by a single frequency ML with vertical linear polarization. The links between SLs presented in Fig. 1 show the mutual couplings (*J*_*ij*_) where the strength of the coupling for each pair of the SLs is symmetric (*J*_*ij*_ = *J*_*ji*_ and *J*_*ii*_ = 0). In a fully connected graph with size of *N*, the number of edges (*N*_edg_) are equal to $({N\atop 2}) = \frac{1}{2}N(N - 1)$ and for the case of an Ising Hamiltonian, there are 2^*N*^ possible spin configurations.

We begin with deriving nonlinear coupled photon rate equations describing the dynamics of a network of injection-locked lasers for the average photon numbers in the left (*n*_*i*L_) and the right (*n*_*i*R_) circular polarization modes, as a function of time. At steady state, i.e., when ${\mathrm{d}}n_{i{\mathrm{R}}}\left( t \right)/{\mathrm{d}}t = {\mathrm{d}}n_{i{\mathrm{L}}}\left( t \right)/{\mathrm{d}}t = 0$ is satisfied, one can derive a mapping (modulo some issues to be discussed later) between Ising spins and the aforesaid steady-state photon numbers at well above threshold, by invoking the minimum gain principle (see Supplementary note 1 for full derivations)^61^. This mapping is as follows:

3 $$\sigma _i = \frac{{\sqrt {n_{i{\mathrm{R}}}} - \sqrt {n_{i{\mathrm{L}}}} }}{{\sqrt {n_{i{\mathrm{R}}} + n_{i{\mathrm{L}}}} }}\,{\mathrm{and}}\;\sigma _j = \frac{{\sqrt {n_{j{\mathrm{R}}}} - \sqrt {n_{j{\mathrm{L}}}} }}{{\sqrt {n_{j{\mathrm{R}}} + n_{j{\mathrm{L}}}} }}$$

The Zeeman term and mutual couplings in Eq. (1) are as follows:

4 $$\lambda _i = 2\sqrt {\frac{Q}{{Q_{\mathrm{M}}}}} \frac{{\sqrt {n_{\mathrm{M}}} }}{{\sqrt {n_{i{\mathrm{R}}} + n_{i{\mathrm{L}}}} }}\chi _i{\mathrm{Cos}}\left( {\varphi _i - \varphi _{{\mathrm{M}}i} - \varphi _{\mathrm{M}}} \right)$$

5 $$J_{ij} = \gamma _{ij}{\mathrm{Cos}}(\varphi _i - \varphi _j - \varphi _{ji})\frac{{\sqrt {n_{j{\mathrm{R}}} + n_{j{\mathrm{L}}}} }}{{\sqrt {n_{i{\mathrm{R}}} + n_{i{\mathrm{L}}}} }},$$

where *Q*_M_ is the ML’s cavity quality factor, $\chi _i$ is the amplitude attenuation coefficients from the horizontal linear polarization of the ML into the *i*th SL. $\gamma _{ij}$ is the amplitude attenuation coefficient for the horizontally polarized signal between *i*th and *j*th SLs which are real and positive when the relative phase difference between two SLs are 0 or ${\pi}$^61,62^. $\varphi _i$ and $\varphi _j$ are the *i*th and *j*th SLs’ phases, respectively. $\varphi _{\mathrm{M}}$ is the ML’s phase, and $\varphi _{{\mathrm{M}}i}$ and $\varphi _{ji}$ are the acquired phases from the *i*th SL locked to ML and *i*th SL to *j*th SL, respectively.

The Ising problem’s solution, i.e., the binary spin configuration, emerges spontaneously at steady state where each spin (defined as a function of *n*_*i*L_ and *n*_*i*R_ above) saturate to binary discrete levels. This behavior can be quantitatively explained via the minimum gain principle, through a natural polarization mode competition enforced by cross-gain saturation rule^61,62^ to minimize Supplementary eq. 6, as discussed in the Supplementary note 1.

In a single mode laser cavity, the bandwidth of the gain medium contains a broad range of modes. However, only one mode gets the chance to amplify and represent the output of the single mode laser. The cavity also has a natural loss which can be varied for different cavity modes. Furthermore, the only mode that lases as the single mode laser, is the mode with the lowest loss in the cavity due to cross-gain saturation^65–67^. Nevertheless, the coherently coupled SLs oscillate with a specific polarization configuration state that minimizes the total loss of the network. The minimum value of the total loss is equal to the total gain of the network^61,62^. The relationship between the above said minimum gain principle and the ground state of the Ising Hamiltonian is based on a numerical verification and does not stand on a solid physical argument.

### Simulation results

**Fig. 2:**
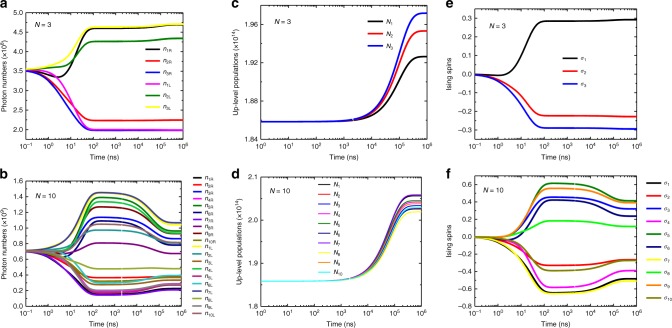
Simulation results. Time dynamic of a network of injection-locked MCF lasers for *N* = 3 (left column) and *N* = 10 (right column). The time evolution analyses are plotted in the log format for **a**, **b** photon number in right and left circular polarizations, **c**, **d** up-level population, and **e**, **f** computed Ising spins. The calculated Ising spin signs for *N* = 3 and *N* = 10 correspond to the ground state spin configuration of introduced Ising Hamiltonians

When the SLs are locked to the ML, by slightly rotating the ML’s vertical polarization the Zeeman term initiates. At the same time by enabling the cross links between SLs, the mutual-coupling terms in the Ising Hamiltonian are turned on. After a short time (defined by the lifetime of the active atoms in the gain medium), the system reaches at the steady state. As we mentioned earlier the left and right circular polarizations are the degree of freedom used in order to determine the sign of Ising spins. If *n*_*i*R_ > *n*_*i*L_ we consider that $\sigma _i = + 1$, and likewise, the spin is set to $\sigma _i = - 1$ if *n*_*i*R_ < *n*_*i*L_ (this convention is applied for $\sigma _j$ as well). We have used a numerical simulation in order to examine the nonlinear coupled laser equations (Supplementary eqs. 3–5) as a CIM, followed by a network of injection-locked MCF lasers. The results of the simulation for *N* = 3 and *N* = 10 SL cores are shown in Fig. 2, for a random choice of the coupling matrix and the Zeeman terms. We set the phase of all SLs to be equal to ML’s phase in the simulation. For *t* < 0, the Zeeman and cross-coupling terms are not turned on. Due to the initialization induced by the only vertically polarized injection-locking signal from the ML, the average photon numbers in two polarization states are equal (*n*_*i*R_ = *n*_*i*L_).

At *t* = 0, the Zeeman and cross-coupling terms are enabled. Immediately afterwards, the average photon numbers in two polarization modes depart from each other due to the effect of polarization control from the ML (Zeeman term) and the mutual couplings among the SLs. Eventually, the system reaches a steady state with a convergence time of about 1 ms controlled by the Yb^3+^ lifetime. The sign of computed Ising spins in Fig. 2e, f are correspond to the ground state spin configuration of the Ising Hamiltonians and we have verified the results with the BFA (See Supplementary note 2 for all parameters used in the simulation).

It should be emphasized that the some of the simulations could not find the exact ground state for some Ising Hamiltonians and they were trapped in local minima (See Supplementary note 3 for an example and summary in Supplementary table (1)). However, one of the great advantage of this CIM could be the very short experimental convergence time that is independent of the number of nodes (only related to the Yb^3+^ lifetime ~1 ms). Therefore, in some cases, the ground state, or approximate ground state, of a very large size Ising Hamiltonian can be found by this proposed CIM in millisecond range time.

### Experimental results

**Fig. 3:**
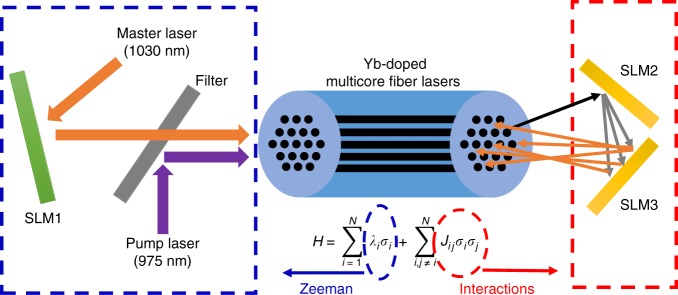
The proposed architecture for the coherent Ising machine (CIM). The injection-locked laser system consists of an Ytterbium doped multicore fiber (MCF) as the slave lasers (SLs), locked to a single frequency master laser (ML). The spatial light modulator 1 (SLM1) is used to implement Zeeman terms via a small change in the vertical polarization state of ML. SLM2 and SLM3 are the elements to program the interaction terms (*J*_*ij*_)

We have performed an all-optical experimental platform to verify and study the performance of the proposed CIM based on a network of injection-locked MCF lasers. Figure 3 represents the concept of proposed optical CIM based on a network of injection-locked MCF lasers.

**Fig. 4:**
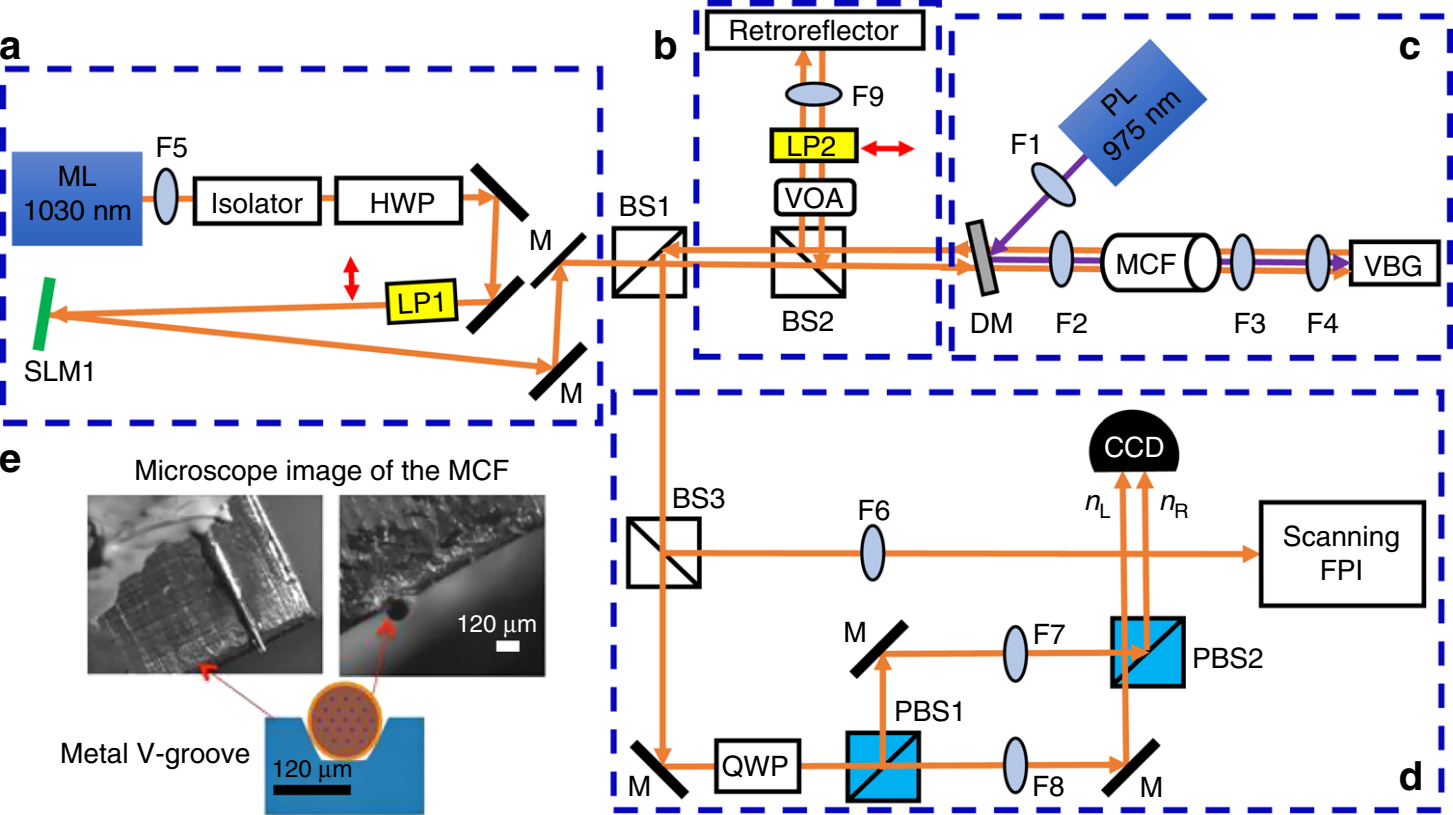
Experimental setup of the coherent Ising machine (CIM) based on a network of injection-locked MCF lasers. **a** The polarization of the single frequency master laser (ML) is set to be vertical using a half-wave plate (HWP) and a linear polarizer (LP1). This block is to implement the Zeeman term using a spatial light modulator (SLM1). **b** This unit is to enable the *J*_*ij*_ terms using a lens F9 and a retroreflector. A variable optical attenuator (VOA) is used to reduce the power of slave lasers (SLs) as the strong feedback can disturb the injection-locking mode. A horizontal LP2 is installed in the pathway of SLs to make sure the Supplementary eqs. 3 and 4 hold. **c** A 975 nm pump laser (PL) is used to pump MCF via a dichroic mirror (DM) which passes 1030 nm and reflect 975 nm. A special made Volume Bragg Grating (VBG) was used as one of the cavity mirror and angled cleaved front facet of the MCF as the second cavity mirror. **d** This section is for polarization measurement through two-polarizing beam splitters (PBS) and a quarter wave-plate (QWP) at 45°. A CCD camera measures the relative intensities between *n*_R_ and *n*_L_. Another purpose of this block is to monitor the injection-locking mechanism through a Fabry–Perot interferometer (FPI). **e** A microscope image of the MCF’s facet on a metal V-groove as the cooling plate. The focal lengths of lens F1, F2, F3, F4, F5, F6, F7, F8, and F9 are +60, +40, +12, +125, +8, +100, +750, +750, and +40 mm, respectively

We have previously reported on successfully injection locking of 19 SLs with a single frequency distributed feedback laser at 1030 nm as the ML^68^. In the present experiment, the SLs were prepared from an MCF composed of proprietary phosphate glasses and highly doped with 6% Yb_2_O_3_ as the gain medium (Made by NP Photonic Inc.). The MCF was cladding pumped by a 975 nm fiber-coupled diode laser. The special design and engineering of the MCF is maintained such that the signal was strongly confined in the cores. Therefore, the cross talks due to the evanescent couplings among the cores are negligible for a short length of MCF^68^. The imaging and focusing lenses, that were used in the system for pumping and injecting the ML, have been selected carefully to reduce the aberration and match with the MCF’s numerical aperture. Figure 4 denotes the experimental setup of the optical CIM. The setup has four main blocks: the Zeeman term initiator (Fig. 4a), the mutual couplings unit (Fig. 4b), the SLs preparation part (Fig. 4c), and finally, the polarization measurement and injection-locking monitoring section (Fig. 4d). The status of injection-locking MCF SLs was being monitored via a Fabry–Perot interferometer (FPI)^68^.

The Zeeman term (*λ*_*i*_) is configured by a programmable diffractive polarizing element like an LCOS spatial light modulator. The polarization state of the injected ML can be set independently for each core of the MCF thanks to the programmable polarization rotation element by using sub-apertures of SLM1. The specific polarization states can be established by setting the retardation coefficients of the pixels composing the different sub-apertures of the SLM1. The total spatial polarization rotation across the ML beam was measured to be 3° (see Supplementary Note 5 for more info).

We implemented two different mutual-coupling schemes, presented in Fig. 4b. The first one is using a flat mirror and a lens in a one-to-one imaging system where the facet of the MCF was inversely imaged onto itself. In this case, only a fixed *J*_*ij*_ matrix with central symmetry could be achieved since retroreflector was not programmable.

**Fig. 5:**
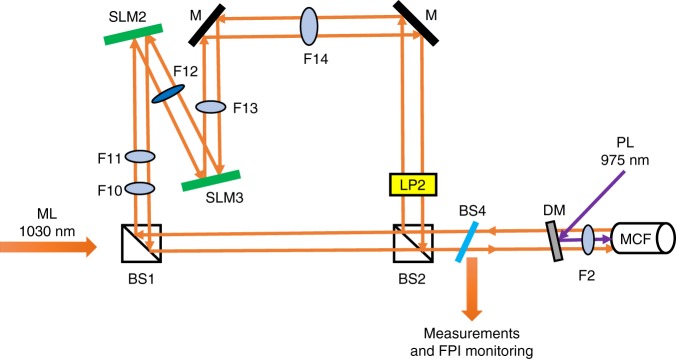
Programmable optical design for the mutual interaction terms. A nonsymmetric optical design is used to implement *J*_*ij*_ elements using two SLMs to control amplitude and connectivity of SLs. All lenses used in this setup are high precision corrected aspheric lens to reduce the aberration (See Supplementary note 4 for more info)

The second design of the mutual coupling used two SLMs. In this case, the connections between SLs can be programmed to control the values of *J*_*ij*_ matrix (Fig. 5). To do so, diffraction gratings are displayed by the SLMs. The frequency and orientation of the gratings define the diffraction direction, the amplitude modulation is responsible for the diffraction amplitude, and the phase of the diffracted beams can be adjusted using the spatial phase of the gratings (lateral shift). To obtain multiple beams from one input SL beam, several gratings are multiplexed. Sub-apertures are defined for each incident SL on the SLM. This system requires two SLMs, one to deflect the beams and the other to restore the angle of incidence to ensure correct injection inside the fiber core.

The SLMs that we used had a compounded diffraction efficiency of 2.13% at 1030 nm. Also, we had to add an extra 50/50 beam splitter (BS) in the optical pathway between SLs (BS1) to make the mutual-coupling symmetric and BS3 (8% reflection ratio) for measurement and FPI monitoring. Hence, we only considered maximum two connections for each SL to other SLs.

The proper optical elements between different SLMs and the SLs are chosen via a primary calculation for Gaussian beams propagation through a nonsymmetric optical system by a so called tensor ABCD law^69,70^ as well as by using a commercially available code Zemax. This investigation was performed in order to match the NA, mode field diameter, radius of curvature and the ray off-set of the SLs beams for the cross-couplings (see Supplementary Note 4).

For the measurement section, we have implemented a simultaneous polarization state readout using a quarter wave-plate at 45°, two polarizing beam splitters and a standard CMOS camera (Fig. 4d). The photon number in left and right circular polarizations which define the effective Ising spins (Eq. (3)), were measured simultaneously by a single camera. The front facet of the MCF was imaged to the camera using a set of positive lenses. F2–F7 and F2–F8 high precision corrected aspheric (up to 14th order to reduce the aberration) lens pairs were used to readout the polarization evolution of the orthogonal polarization states of the CIM as shown in the Fig. 4. The camera was a CMOS pixel array sensitive at near infrared wavelength. We developed a MATLAB scripts to read the relative intensity between the left and right circular polarizations of each core from the camera files.

### Central symmetry coupling

**Fig. 6:**
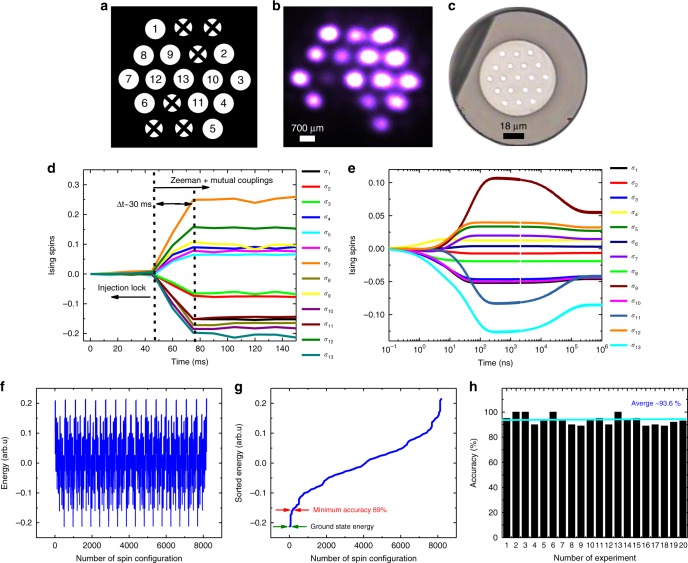
Central symmetry couplings results. **a** A scheme of MCF for *N* = 13 and node connections in an image inversion transformation. The connections are between SL 1–5, 2–6, 3–7, 4–8, 9–11, and 10–12. **b** An image of the MCF’s facet with 16 SLs that have the most stable and uniform power for the experiment while three of SLs cannot be used for the Ising Hamiltonian because of the inverse imaging cross-couplings. This makes the Ising Hamiltonian with size of *N* = 13. **c** A microscope picture from the facet of the Ytterbium doped phosphate glass MCF. **d** The calculated Ising spins evolution based on the measured relative intensities between photon numbers in right and left circular polarizations, detected by a CMOS CCD camera. **e** Simulated Ising spins based on the experimental data that we fed to the Ising solver code and excellent agreement with **d** in terms of spin signs. Both experiment and MCF simulation found the ground state spin configuration for the Ising Hamiltonian. **f** Computed Ising energy values for all possible spin configurations (2^13^) using a brute-force algorithm (BFA). **g** Sorted Ising energy values from **f** versus number of spin configurations. **h** Average accuracy of the optical coherent Ising machine (CIM) for that particular Ising Hamiltonian with *N* = 13, was calculated to be 93.6% of actual value of the ground state Ising Hamiltonian. The minimum accuracy that was performed by the experiment was 89%. The CIM relaxed to the exact ground state four times out of 20 times experimentations

For the mutual coupling terms (*J*_*ij*_) using a reflector mirror and a lens, any MCF slave laser core is coupled to the geometrically symmetric core in the 19-core array. Although 19 cores are available, we only employed 13 cores of the MCF because three cores did not have good injection-locking quality and three cores were not able to connect to any SL due to the symmetric inversion transformation (See Fig. 6a, b).

Considering reflection and coupling losses, a maximum of 25% of total power would couple back to the MCF cores. We observed experimentally that this amount of feedback can disturb the states of the injection-locking lasers and making the system unstable and out of locking eventually. For that reason, we placed an attenuator to couple a smaller amount of light back to the MCF cores. Typically, we allow a maximum of ~5% power coupling to take place. Figure 6d shows the experimental results of the optical CIM for *N* = 13 SLs.

For this experiment, the MCF was pumped at 3.8 W (the threshold pump power for lasing 13 SLs), and injection of ~183 mW from the ML into the inner cladding of the MCF. Initially, the SLM1 was set at constant retardation, and the mutual-coupling unit was mechanically blocked by a slow shutter (*t* < 50 ms in Fig. 6d left side). Both the mutual-coupling shutter and the Zeeman terms were abruptly turned on at *t* = 0 (*t* = 50 ms in Fig. 6d), and the polarization states were recorded as a function of time until steady state was performed (*t* > 77 ms in Fig. 6d right side).

The convergence time of the steady state for the polarization states were limited to the slow speed of the mechanical shutter, the camera readout time and the analog–digital data conversion. The difference between photon number in right and left circular polarizations for each SL was observed to move towards positive spin (+1) or negative spin (−1) as expected (Eq. (3)). The signal-to-noise ratio of the photon numbers at the camera readout was high enough to easily distinguish between negative and positive Ising spins for each SL. Figure 6e shows the numerical simulation of the CIM based on the experimental data that we obtained for the Zeeman and mutual-coupling terms (see Supplementary note 6 for the specific choices of the coupling matrix and Zeeman terms we made, and the experimental data).

We compared this result with a BFA and it confirms that the MCF simulation found the ground state of the Ising Hamiltonian. We repeated the experiment 20 times (turning on and off the Zeeman and mutual coupling terms) in a 20 s time frame duration. Four times the optical CIM found the exact ground state. For the 16 other cases, the system converged toward local minima with minimum and maximum accuracy of 89 and 95% of the ground state energy, respectively (Fig. 6f, g). Figure 6h denotes the accuracy versus number of experimental trial.

The optical CIM for this particular Hamiltonian, maintained the average accuracy of ~93%. There are various reasons that could explain why the system did not always fall in the ground state. One explanation is the external disturbances on the SLs’ cavity, such as airflow and vibration, which perturbed the quality of the injection-locking network. Fundamentally, the noise and fluctuations from the ML or the pump could also effect on the results^61^.

### General coupling matrices

**Fig. 7:**
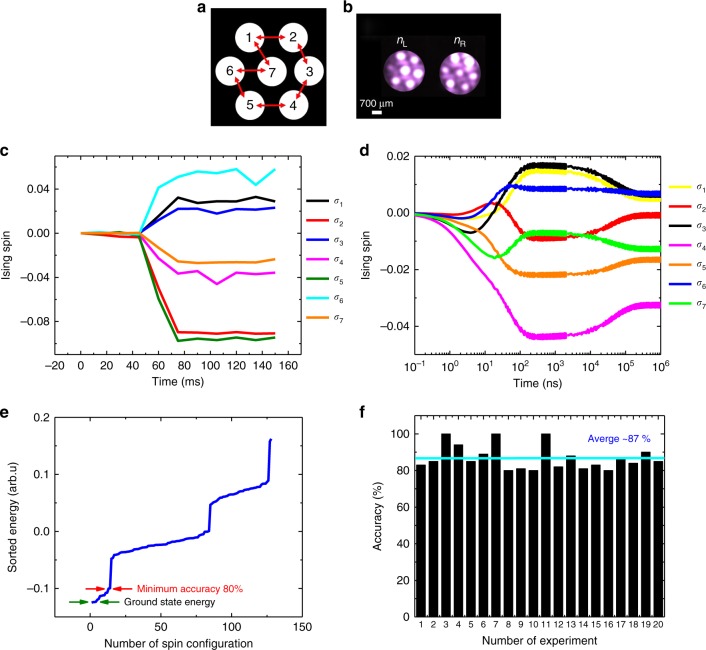
One-dimension (1D) Ising Hamiltonian with *N* = 7. **a** A ring grating design for the mutual coupling with maximum two connections. **b** A picture of the facet from CCD when the Zeeman terms and mutual couplings were enabled. The intensity (brightness) of right and left circular polarizations change as the function of time. **c** Experimental Ising spin evolution as the function of time. **d** Simulation result of the proposed CIM and with a good agreement with Ising spin signs in **c**. **e** Sorted energy value versus all spin configurations (2^7^). **f** Accuracy versus number of experiment. The overall average accuracy of the CIM for this Ising Hamiltonian was 87%

We have performed the experiment for some other Ising Hamiltonians based on the optical design Using the diffraction from two SLMs to control the *J*_*ij*_ terms. For the mutual couplings terms, three gratings with different connectivity and modulation amplitude were uploaded to the SLMs. Figure 7a describes the first connectivity that was implemented between the SLs, and where each SL was only connected to two other SLs. Figure 7b is a picture of the facet of the MCF at the time when the Ising Hamiltonian was turned on. It can be seen that the brightness between *n*_R_ and *n*_L_ are different. The brightness’s change as the function of time until the system reaches to the steady state after 1 ms ($\frac{{{\mathrm{d}}n_{i\mathrm{R}}}}{{{\mathrm{d}}t}} = 0$ & $\frac{{{\mathrm{d}}n_{i\mathrm{L}}}}{{{\mathrm{d}}t}} = 0$ but $\sqrt {n_{i\mathrm{R}}} - \sqrt {n_{i\mathrm{L}}} \,\,\ne \,\, 0$). Figure 7c, d denote the Ising spin signs based on the experimental measurement and MCF simulation, respectively. Figure 7e shows the sorted Ising energy versus the number of spin configuration (2^7^ spin configurations) produced by the BFA. Figure 7f is the accuracy of computed Ising energy based on measured Ising spin configurations versus the number of experiments. (see Supplementary note 6 for the experimental data).

**Fig. 8:**
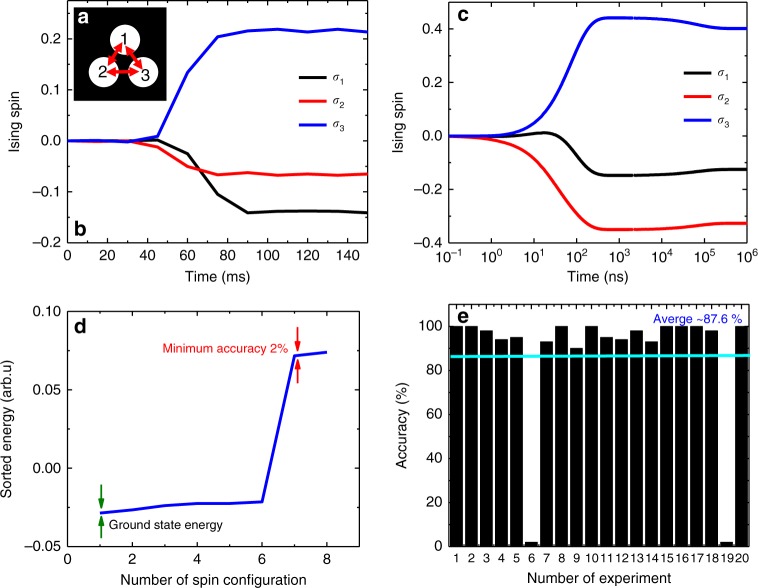
Ising Hamiltonian with *N* = 3. **a** Fully connected grating design for the mutual coupling matrix. **b** Experimental Ising spin evolutions as the function of time. **c** Simulation result of the given Ising Hamiltonian. The CIM simulation found exact ground state. **d** Sorted Ising energy values for the eight possible configurations. **e** Accuracy versus number of experiment. The overall average accuracy of the CIM for the designed Ising Hamiltonian in **a** was 87.6%. The CIM found the ground state 8 times out of 20 times experiment and trapped twice to an accuracy of 2%

**Fig. 9:**
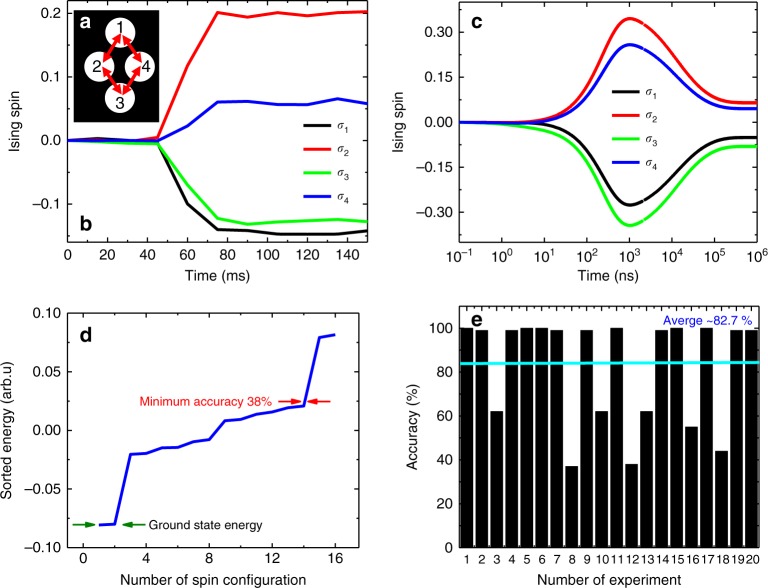
Square lattice Ising Hamiltonian with *N* = 4. **a** Lattice connectivity design for the mutual coupling matrix. **b** Experimental Ising spin evolutions as the function of time. **c** Simulation result of the given Ising Hamiltonian. The CIM simulation found exact ground state. **d** Sorted Ising energy values for the 16 possible configurations. **e** Accuracy versus number of experiment. The overall average accuracy of the CIM for the designed Ising Hamiltonian in **a** was 82.7%. The CIM found the ground state 6 times out of 20 times experiment and trapped twice to an accuracy of 38%

We performed the experiment for another example with size of *N* = 7 (see Supplementary Note 7) and the CIM found the ground state with overall average of ~90% accuracy. Figures 8 and Fig. 9 denote two Ising Hamiltonian instances with size of *N* = 3 and *N* = 4, respectively (see Supplementary Note 6 for the experimental data).

## Discussion

We investigated both theoretically and experimentally an all-optical CIM based on a network of injection-locked MCF lasers. The numerical simulation of the system can find the ground state Ising spin configuration consistent with the BFA results. Although, we should note that in some cases, the numerical simulation of the injection-locking network did not find the ground state. As proof-of-principle, we performed several experiments for different Ising Hamiltonians with size of *N* = 3, *N* = 4, *N* = 7, and *N* = 3, repeating the experiment 20 times each. Table 1 denotes a summary of the experimental results:

**Table. 1:** Summary of experimental results. *N* and *N*_edg_ stand for number of Ising nodes and number of edges, respectively. NTG stands for number of times that the experiment trapped to the ground state out of 20 times experiment for each case. *A*_min_ and *A*_ave_ denote the minimum and average accuracies, respectively

*N*	*N*_edg_	NTG	*A*_min_ (%)	*A*_ave_ (%)
3	3	8	2	87
4	4	6	38	82
7	7	4	80	90
13	6	4	90	93

We attribute the fluctuations observed in the experimental system to some macroscopic effects such as vibration or airflow in the cavity, changing the length of the cavity because of temperature fluctuations in the lab or any other effect that can change the status of injection-locking system. The proposed optical CIM converges to the result in 1 ms and it does not increase as the problem size increases.

Increasing the number of cores in the MCF could scale the Ising Hamiltonian if stable injection locking is maintained. Hypothetically, increasing the bandwidth of the injection locking is one of the key element in order to scale the CIM to higher nodes. Increasing the number of cores, needs a higher pump power. In this case the cooling of the MCF should be also well controlled in order to keep the length of the cavity constant. Other macroscopic affects such as thermal fluctuations and vibrations can disturb the cavity of SLs and they need to be taken in account. The other important key element to scale the CIM is implementing SLMs with high diffraction efficiency and low loss. This allows increasing number of edges in the mutual-coupling matrix. However, each SL’s energy is reduced by the of square of the number of connections which reduces the feedback to the MCF. (We experimentally found in our system that the feedback power ratio needs to be <5% and >1%). For instance, replacing the current SLMs (2.13% diffraction efficiency) with optimized SLMs for 1030 nm wavelength (~94% diffraction efficiency), allows us to implement fully connected Ising Hamiltonian up to *N* = 95 nodes and $({N\atop 2})$ ~ 4465 edges (see Supplementary note 8 for more details). Furthermore, using the CCD camera with higher pixel depth can result in principle, to detect more accurately the variations between *n*_R_ and *n*_L_. In other word, the lower bound limitation for the feedback power ratio (1%), can even be pushed to be smaller which would permit us to implement the CIM with higher nodes and edges (see Supplementary note 8).

According to Eqs. (4) and (5), the Zeeman term and mutual couplings are dependent on the photon numbers (*n*_*i*L_ and *n*_*i*R_) which indicates that the described mapping of the CIM to the Ising Hamiltonian is dependent on the solution of the specific Ising instance to be solved. This can be a major issue to program the CIM to solve any arbitrary Ising Hamiltonian specially when we want to feed a hard instance of an NP-hard problem into the CIM. However, we can approximate the ratios involving *n*_*i*L_ and *n*_*i*R_ in Eqs. (4) and (5) to one since the magnitudes of the total energy in the SLs and ML can be taken to be almost equal, i.e., $n_{i{\mathrm{L}}} + n_{i{\mathrm{R}}} = n_{j{\mathrm{L}}} + n_{j{\mathrm{R}}}$ for all *i* and *j*.

However, despite the above issues of the solution-dependent mapping, the original proposed CIM based on the injection-locked lasers^61^ is an interesting nonlinear coupled optical system worthwhile of exploration as a platform for analog computation. The system seeks a physical energy minimum driven by its Lagrangian. The challenge will be to find a computationally hard problem that can be mapped into that physical Lagrangian. It however is intriguing that despite these purportedly imprecise mapping, the CIM finds the ground state or at least a local minimum close to the ground state, for problems of small sizes.

Future work must entail investigation of the sensitivity and robustness of the proposed CIM based on a network of injection-locked MCF lasers. It should be emphasized that this CIM proposal should not be construed as a claim to solve NP-Hard problems efficiently. As such, it will be important to characterize the approximation ratios (to the cost of the optimal solution) attained by this solver, and compare that to those attained by the best-known (digital) PTAS for the problems at hand.

## Supplementary information

Supplementary Information

## Data Availability

The data that support the plots within this paper and other findings of this study are available from the corresponding author on reasonable request.

## Code availability

Simulation and data analyses codes are available from the corresponding author on reasonable request.
