# Supplementary material for: A single shot coherent Ising machine based on a network of injection-locked multicore fiber lasers
Source: Nat Commun. 2019 Aug 6;10:3516. doi: 10.1038/s41467-019-11548-4 (PMC6684570; doi:10.1038/s41467-019-11548-4)
Supplement: Supplementary file 1 — Supplementary Information [file 41467_2019_11548_MOESM1_ESM.pdf]

Supplementary Information

A single shot coherent Ising machine based on a network of injection-locked multicore fiber lasers

Masoud Babaeian[1,2,*], Dan T. Nguyen[1,3], Veysi Demir[4], Mehmetcan Akbulut[1], Pierre-A. Blanche[1], Yushi Kaneda[1], Saikat Guha[1] , Mark A. Neifeld[1,5], and N. Peyghambarian[1]

[1] *College of Optical Sciences, University of Arizona, Tucson AZ 85721, USA*

[2] *Department of Physics, University of Arizona, Tucson AZ 85721, USA*

[3] *Corning Research and Development Corporation, Corning, NY 14831, USA*

[4] *Currently at ASML Corp. Wilton, CT 06897, USA*

[5] *Department of Electrical and Computer Engineering, University of Arizona, Tucson AZ 85721, USA*

*Babaeian@physics.arizona.edu

Supplementary Note 1: Theoretical Background and Mapping Protocol to the Ising Hamiltonian.

Here we discuss a formal mechanism that brings a relationship between nonlinear coupled rate equations of a network of injection-locked lasers and an Ising Hamiltonian defined as:

$$H = \sum_{i,j\neq i}^{N} J_{ij}\sigma_i\sigma_j + \sum_{i=1}^{N} \lambda_i\sigma_i \tag{1}$$

where H is the Ising Hamiltonian, J_{ij} and λ_i are the mutual couplings and the Zeeman term respectively. σ_i denotes the Ising spin for the ith site. We shall start from the Heisenberg-Langevin equation of a laser cavity, to describe the dynamics of an injection-locked laser system[1,2]:

$$\frac{\mathrm{d}A_{i\Delta}(t)}{\mathrm{d}t} = -\mathrm{i}\omega_{\mathrm{s}}A_{i\Delta}(t) - \frac{1}{2}\left(\frac{\omega}{Q} - \frac{\omega}{r_0^2}(g_i - \mathrm{i}D)\right)A_{i\Delta}(t) + \sqrt{\frac{\omega}{Q}}A_{\mathrm{M}i} + \frac{\omega}{2Q}\sum_{j\neq i}^{N}\tilde{A}_{ij\Delta} + A_{QN} \tag{2}$$

where $A_{\mathrm{M}i}$ is the master laser's (ML) field and $A_{i\Delta}(t)$ is the complex field operator of the ith slave laser (SL). Δ denotes the mode of the field (or phase) in left (L) or right (R) circular polarization modes. ω and ω_{s} are the lasing frequency of the ML and the SL respectively. $\tilde{A}_{ij}$ is the coupling field from laser j onto laser i and A_{QN} denotes the quantum fluctuation. Q is the cavity quality factor, r_0 and D are the non-resonant refractive index and the nonlinear dispersion respectively. $G_i = (\omega/r_0^2)g_i$ is the net gain. Inserting photon number operator which is defined as $n(t) = A^{\dagger}(t)A(t)$, in Supplementary equation (2) for left and right circular polarization modes and applying mean field approximation, results[3,4]:

$$\frac{\mathrm{d}n_{i\mathrm{L}}}{\mathrm{d}t} = -\left[\frac{\omega}{Q} - G_i\right] n_{i\mathrm{L}} + 2\sqrt{\frac{\omega}{Q}}\sqrt{\frac{\omega}{Q_{\mathrm{M}}}}(\psi_i + \chi_i)\sqrt{n_{i\mathrm{L}}}\sqrt{n_{\mathrm{M}i}}\mathrm{Cos}(\varphi_{i\mathrm{L}} - \varphi_{\mathrm{M}i} - \varphi_{\mathrm{M}})$$
$$+ \frac{\omega}{Q}\sum_{j\neq i}\gamma_{ij}\sqrt{n_{i\mathrm{L}}}[\sqrt{n_{j\mathrm{R}}}\,\mathrm{Cos}(\varphi_{i\mathrm{L}} - \varphi_{j\mathrm{R}} - \varphi_{ji}) - \sqrt{n_{j\mathrm{L}}}\mathrm{Cos}(\varphi_{i\mathrm{L}} - \varphi_{j\mathrm{L}} - \varphi_{ji})] \quad (3)$$

$$\frac{\mathrm{d}n_{i\mathrm{R}}}{\mathrm{d}t} = -\left[\frac{\omega}{Q} - G_i\right] n_{i\mathrm{R}} + 2\sqrt{\frac{\omega}{Q}}\sqrt{\frac{\omega}{Q_{\mathrm{M}}}}(\psi_i - \chi_i)\sqrt{n_{i\mathrm{R}}}\sqrt{n_{\mathrm{M}i}}\mathrm{Cos}(\varphi_{i\mathrm{R}} - \varphi_{\mathrm{M}i} - \varphi_{\mathrm{M}})$$
$$- \frac{\omega}{Q}\sum_{j\neq i}\gamma_{ij}\sqrt{n_{i\mathrm{R}}}[\sqrt{n_{j\mathrm{R}}}\,\mathrm{Cos}(\varphi_{i\mathrm{R}} - \varphi_{j\mathrm{R}} - \varphi_{ji}) - \sqrt{n_{j\mathrm{L}}}\mathrm{Cos}(\varphi_{i\mathrm{R}} - \varphi_{j\mathrm{L}} - \varphi_{ji})] \quad (4)$$

where Q_{M} is the ML's cavity quality factor, ψ_i and χ_i are the amplitude attenuation coefficients from the vertical and horizontal linear polarization of the ML into the ith SL respectively. γ_{ij} is the attenuation coefficient for the horizontally polarized signal between i^{th} and j^{th} SLs. $\varphi_{i\Delta}$ and $\varphi_{j\Delta}$ are the ith and j^{th} SLs' phases for left and right circular polarizations respectively. $\varphi_{\mathrm{M}i}$ and φ_{ji} are the acquired phases from the ith SL locked to ML and i^{th} SL to j^{th} SL respectively and φ_{M} is the ML's phase. We have approximated (mean field approximation) all the complex optical fields in Supplementary equation (2) as: $A_{i\Delta} = \sqrt{n_{i\Delta}}e^{-i(\omega t+\varphi_i)}$, $A_{j\Delta} = \sqrt{n_{j\Delta}}e^{-i(\omega t+\varphi_j)}$, $A_{\mathrm{M}i} = \sqrt{(\omega/Q_{\mathrm{M}})n_{\mathrm{M}i}}e^{-i(\omega t+\varphi_{\mathrm{M}})}$ and disregarded the quantum fluctuations. We also considered the coupling filed as $\tilde{A}_{ij} = (A_{j\mathrm{R}} - A_{j\mathrm{L}})\gamma_{ij}e^{-\mathrm{i}\varphi_{ji}}$.

The rate equation of the up-population ($N_i(t)$) for an optically pumped SL is defined as[5-9]:

$$\frac{\mathrm{d}N_i(t)}{\mathrm{d}t} = \frac{N_T - 2N_i(t)}{\tau_{\mathrm{sp}}}P_i - \frac{N_i(t)}{\tau_{\mathrm{sp}}} - G_i[n_{i\mathrm{R}}(t) + n_{i\mathrm{L}}(t) + 2] \quad (5)$$

where N_T is the total optical population, P_i is the pump level for i^{th} SL and τ_{sp} is the spontaneous emission lifetime. The nonlinear dynamic laser equations, Supplementary equations (3-5) are linked by $G_i = \delta N_i\,(t)/\tau_{\mathrm{sp}}$ where δ is the coupling efficiency of the spontaneous emission into a lasing mode[3]. We note that the quantum noise operators are disregarded in set of Supplementary equations (3-5). When the injection-locked laser system is prepared, the Zeeman terms and mutual couplings in the Ising Hamiltonian can be implemented by slightly rotating the ML's vertical polarization and enabling the cross links between SLs respectively. The system reaches to a steady state after a short time which is defined by the lifetime of the active atoms in the gain medium. At the steady state $\mathrm{d}n_{i\mathrm{R}}\,(t)/\mathrm{d}t = \mathrm{d}n_{i\mathrm{L}}\,(t)/\mathrm{d}t = \mathrm{d}N_i\,(t)/\mathrm{d}t = 0$ must hold. Thus, from this condition, we can derive the total gain as the following ($G_{\mathrm{Tot}} = \sum_{i=1}^{N} G_i$):

$$\begin{aligned}\frac{Q}{\omega}G_{\mathrm{Tot}} = -1 &+ 2\sqrt{\frac{Q}{Q_\mathrm{M}}}\sum_{i}^{N}\psi_i \mathrm{Cos}(\varphi_i - \varphi_{\mathrm{M}i} - \varphi_\mathrm{M})\sqrt{n_\mathrm{M}}\,\frac{\sqrt{n_{i\mathrm{R}}}+\sqrt{n_{i\mathrm{L}}}}{n_{i\mathrm{L}}+n_{i\mathrm{R}}} \\ &- 2\sqrt{\frac{Q}{Q_\mathrm{M}}}\sum_{i}^{N}\chi_i \mathrm{Cos}(\varphi_i - \varphi_{\mathrm{M}i} - \varphi_\mathrm{M})\sqrt{n_\mathrm{M}}\,\frac{\sqrt{n_{i\mathrm{R}}}-\sqrt{n_{i\mathrm{L}}}}{n_{i\mathrm{L}}+n_{i\mathrm{R}}} \\ &- \sum_{i,j\neq i}^{N}\gamma_i \mathrm{Cos}(\varphi_i - \varphi_j - \varphi_{ji})\frac{\sqrt{n_{j\mathrm{R}}+n_{j\mathrm{L}}}}{\sqrt{n_{i\mathrm{R}}+n_{i\mathrm{L}}}}\frac{\sqrt{n_{i\mathrm{R}}}-\sqrt{n_{i\mathrm{L}}}}{\sqrt{n_{i\mathrm{R}}+n_{i\mathrm{L}}}}\frac{\sqrt{n_{j\mathrm{R}}}-\sqrt{n_{j\mathrm{L}}}}{\sqrt{n_{j\mathrm{R}}+n_{j\mathrm{L}}}}\end{aligned} \tag{6}$$

To obtain Supplementary equation (6), we have set $\varphi_{i\mathrm{R}} = \varphi_{i\mathrm{L}} \equiv \varphi_i$ and $\varphi_{j\mathrm{R}} = \varphi_{j\mathrm{L}} \equiv \varphi_j$ in Supplementary equations (3) and (4). We also assumed all SLs receive equal energy from the ML ($n_{\mathrm{M}i} = n_{\mathrm{M}j} = n_\mathrm{M}$) as well as from the pump. The last two terms of left-hand-side of Supplementary equation (6) are equivalent to the Ising Hamiltonian if we introduce the Ising spin as:

$$\sigma_i = \frac{\sqrt{n_{i\mathrm{R}}} - \sqrt{n_{i\mathrm{L}}}}{\sqrt{n_{i\mathrm{R}} + n_{i\mathrm{L}}}} \quad \text{and} \quad \sigma_j = \frac{\sqrt{n_{j\mathrm{R}}} - \sqrt{n_{j\mathrm{L}}}}{\sqrt{n_{j\mathrm{R}} + n_{j\mathrm{L}}}} \tag{7}$$

where the Zeeman term and mutual couplings are corresponded to the following mathematical forms with respect to Supplementary equation (1):

$$\lambda_i = 2\sqrt{\frac{Q}{Q_\mathrm{M}}} \frac{\sqrt{n_\mathrm{M}}}{\sqrt{n_{i\mathrm{R}} + n_{i\mathrm{L}}}} \chi_i \mathrm{Cos}(\varphi_i - \varphi_{\mathrm{M}i} - \varphi_\mathrm{M}) \tag{8}$$

$$J_{ij} = \gamma_{ij} \mathrm{Cos}(\varphi_i - \varphi_j - \varphi_{ji}) \frac{\sqrt{n_{j\mathrm{R}} + n_{j\mathrm{L}}}}{\sqrt{n_{i\mathrm{R}} + n_{i\mathrm{L}}}} \tag{9}$$

Here, we consider $\sigma_i = +1$ and $\sigma_i = -1$ if $n_{i\mathrm{R}} > n_{i\mathrm{L}}$ and $n_{i\mathrm{R}} < n_{i\mathrm{L}}$ respectively (this convention is applied for σ_j as well).

Supplementary Note 2: Simulation Parameters Used in Coherent Ising Machine Solver for *N*=3 and *N*=10 Node

The nonlinear coupled injection-locked lasers system, Supplementary equations (3-5) have been solved using 4th order Runge Kutta method. We assume the ML's phase and SLs' phases are equal at the time when the network of injection-locked lasers is implemented (the Ising Hamiltonian is still not enabled). The Zeeman terms (λ_i) and mutual couplings (J_{ij}) are the raw input data to the coherent Ising machine (CIM) simulator. The code computes the photon number in left and right circular polarizations as the function of time. The Ising spins then are calculated as the function of time based on Supplementary equation (7). The sign of Ising spins at the steady state is correspond to the Ising spin configuration that we search for. Furthermore, we calculate the exact ground state Ising spin configuration, using a brute-force algorithm (BFA) to compare the results with CIM solver.

For the Ising Hamiltonian with *N*=3 nodes the Zeeman and mutual coupling terms are assumed to be the following:

$$\lambda_{1\times 3} = \begin{pmatrix} 0.01 & 0.02 & 0.03 \end{pmatrix} \tag{10}$$

$$J_{3\times 3} = \begin{pmatrix} 0 & 0.1 & 0.12 \\ 0.1 & 0 & 0.05 \\ 0.12 & 0.05 & 0 \end{pmatrix} \tag{11}$$

And for the Ising Hamiltonian with size of *N*=10:

$$\lambda_{1\times 10} = \frac{1}{100}\begin{pmatrix} 3 & 2 & 0.7 & 1 & 0.6 & 1.2 & 4 & 0.2 & 0.5 & 0.9 \end{pmatrix} \tag{12}$$

$$J_{10\times 10} = \frac{1}{100}\begin{pmatrix} 0 & 2 & 0.1 & 0.3 & 5 & 4 & 0 & 0.9 & 1.5 & 1.7 \\ 2 & 0 & 6 & 1.3 & 0 & 0.2 & 0.1 & 0 & 3 & 0.1 \\ 0.1 & 6 & 0 & 2 & 0 & 0.9 & 8 & 3 & 0 & 0.4 \\ 0.3 & 1.3 & 2 & 0 & 3 & 4 & 0 & 0.9 & 0.1 & 0 \\ 5 & 0 & 0 & 3 & 0 & 7 & 2 & 0.1 & 0 & 2 \\ 4 & 0.2 & 0.9 & 4 & 7 & 0 & 0.9 & 0 & 1 & 0 \\ 0 & 0.1 & 8 & 0 & 2 & 0.9 & 0 & 2 & 4 & 0 \\ 0.9 & 0 & 3 & 0.9 & 0.1 & 0 & 2 & 0 & 0 & 0.3 \\ 1.5 & 3 & 0 & 0.1 & 0 & 1 & 4 & 0 & 0 & 5 \\ 1.7 & 0.1 & 0.4 & 0 & 2 & 0 & 0 & 0.3 & 5 & 0 \end{pmatrix} \quad (13)$$

The CIM simulator based on the injection-locking multicore fiber lasers, for both Ising Hamiltonians found the ground state and the results are confirmed with BFA (Fig. 3e and Fig. 3f). The cavity lifetime and lifetime of the active atoms in the gain medium (Yb^{3+}) are assumed to be 1 ns and 1 ms respectively. The pump level was set to 3.5 (arb.u.). The initial conditions and attenuation factors (χ_i and γ_{ij}) were chosen such that a weak injection-locking system performed. The strength of the cross couplings and the Zeeman terms were also selected to be weak (i.g. a few percent like 10% of light coupled among SLs). Maintaining weak injection-locking and weak cross links in the Ising Hamiltonian is very important in order to obtain the ground state of the Ising Hamiltonian in the CIM simulator.

We have performed the simulation up to N=19 nodes and for all cases the simulation relaxed to the ground state Ising Hamiltonian. However, as we mentioned the CIM simulation cannot find the exact ground state for some cases and the computed Ising spins are correspond to a local minimum. This happens mainly when the strength of cross couplings or the Zeeman terms are considered to be strong compare to the attenuation factors (χ_i and γ_{ij}). It also may be due to the lack of quantum noise in the simulation[3,4].

Supplementary Note 3: Local Minimum Rather Than Ground State

The following Ising Hamiltonian is an example where the CIM simulator trapes in a local minimum:

$$\lambda_{1\times3} = \begin{pmatrix} 3 & 7 & 2 \end{pmatrix} \tag{14}$$

$$J_{3\times3} = \begin{pmatrix} 0 & 3 & 0.12 \\ 3 & 0 & 5 \\ 0.12 & 5 & 0 \end{pmatrix} \tag{15}$$

Supplementary Figure 1 denotes the results for this Ising Hamiltonian.

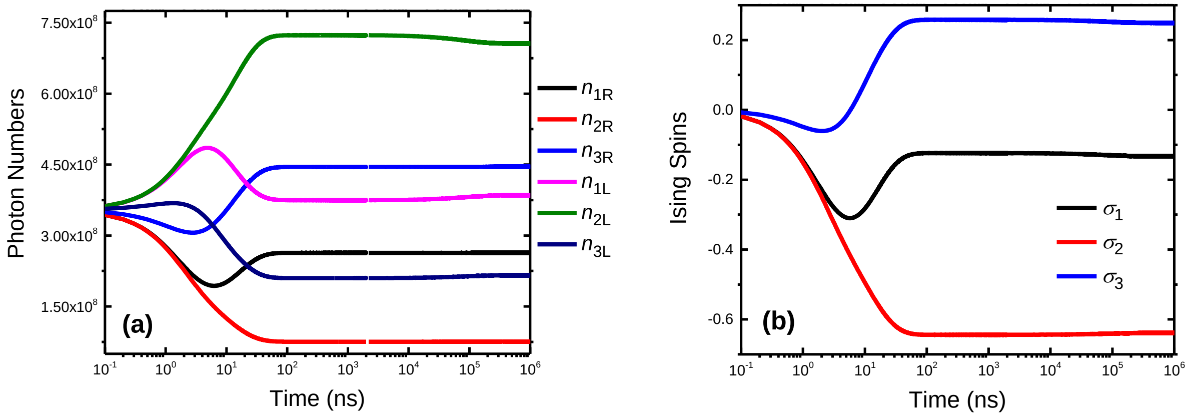

Supplementary Figure 1. Numerical simulation of the Ising Hamiltonian with *N*=3 nodes. a Computed photon number in left (L) and right (R) circular polarizations as the function of time. **b** Calculated Ising spins based on Supplementary equation (7).

The signs of Ising spins at the steady state for σ_1, σ_2 and σ_3 are -1, -1 and +1 respectively (Supplementary Figure 1b). The calculated Ising spins based on BFA are +1, -1 and +1 for σ_1, σ_2 and σ_3 respectively. Supplementary Table 1 summarizes the Ising spins and the expectation values of the Ising Hamiltonian ($\langle H \rangle$) based on CIM simulation and BFA.

Method	σ_1	σ_2	σ_3	$\langle H \rangle$ (arb.u.)
CIM	-1	-1	+1	-12.24
BFA	+1	-1	+1	-17.76

Supplementary Table 1. Numerical results for CIM simulation versus BFA.

As it can be notices the CIM simulation for the mentioned Ising Hamiltonian lied in a local minimum.

Supplementary Note 4: Optical Design for The Mutual Interactions and ABCD Law

In this section we discuss the optical design (Fig. 5) that is used for the implementation of the mutual couplings using two spatial light modulators (SLMs). The SLs are prepared via a Yb-doped phosphate multicore fiber (MCF)[10]. The radius of each core (or basically each SL) is 1.9 µm with a core-to-core spacing of 13 µm. The outer and inner cladding diameters are 120 µm and 72 µm respectively. The numerical aperture (NA) of the cores is 0.15. The Rayleigh length (Z_R) for each SL at wavelength of λ=1030 nm is roughly 11 µm ($Z_R=\pi r^2/\lambda$, where r is the beam radius). Such a short Rayleigh length does not allow experimentally to implement core to core or to some cores connections properly.

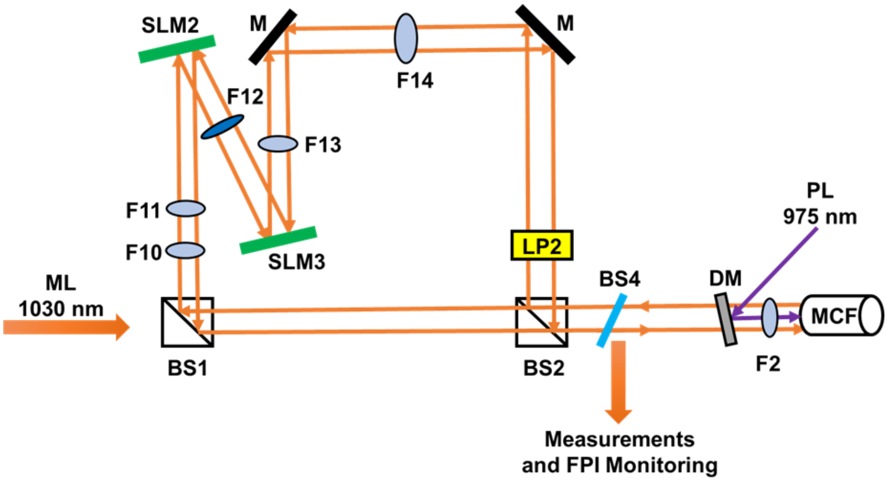

Supplementary Figure 2. Programmable optical design for the mutual interaction terms. A non-symmetric optical design is used to implement J_{ij} elements using two SLMs to control amplitude and connectivity of SLs. All lenses used in this setup are high precision corrected aspheric lens to reduce the aberration. The focal lengths of lens F2, F10, F11, F12, F13, F14 are +40 mm, +40 mm, +4 mm, -1000 mm, +20 mm, +300 mm respectively. The distance between pair lens F2-F10, F10-F11, F11-F12, F12-F13, F13-F14, F14-F2 are d_1=700 mm, d_2=44 mm, d_3=90 mm, d_4=80 mm, d_5=320 mm and d_6=700 mm respectively.

The beams need to be well collimated and separated from each other at the surface of the SLMs and re-injected back to the cores based on the type of the Ising Hamiltonian. Nevertheless, we can first collimate the overlapped SLs' beams via F2 lens (Supplementary Figure 2) and image it to a far filed via an imaging system (F10 and F11 in Supplementary Figure 2). This technique allows the beams become separated from each other at the surface of the SLMs (particularly SLM2 and SLM3 in Supplementary Figure 2) and stay almost collimated till the other optical lenses (F13, F14 and F2 in Supplementary Figure 2) re-inject the light back to the proper cores with appropriate NA, spot size, radius of curvature (*R*) and corrected off-set ray beam tracing. Therefore, a proper optical design is necessary to satisfy these requirements.

The focal lengths (*F*) and the distances (*d*) between the optical elements provided in Supplementary Figure 2 are selected via a formal matrix method that so called tensor ABCD law for Gaussian beams propagation through a non-symmetric system[11,12]:

$$\begin{pmatrix} q_2 \\ 1 \end{pmatrix} = \begin{pmatrix} A & B \\ C & D \end{pmatrix} \begin{pmatrix} q_1 \\ 1 \end{pmatrix} \tag{16}$$

where q_2 and q_1 are the optical parameters for the final and initial points respectively and are defined as the following:

$$\frac{1}{q_2} = \frac{1}{R_2} + \frac{\mathrm{i}\lambda}{\boldsymbol{\pi} r_2^2} \tag{17}$$

$$\frac{1}{q_1} = \frac{1}{R_1} + \frac{\mathrm{i}\lambda}{\boldsymbol{\pi} r_1^2} \tag{18}$$

where R_1 and R_2 are the radius of curvature of the beam for the initial and final points respectively. To compute the final NA and final off-set (y_2) ray we can use:

$$\begin{pmatrix} y_2 \\ \mathrm{NA}_2 \end{pmatrix} = \begin{pmatrix} A & B \\ C & D \end{pmatrix} \begin{pmatrix} y_1 \\ \mathrm{NA}_1 \end{pmatrix} \tag{19}$$

Thus the transfer matrix for the optical system in Supplementary Figure 2 can be written as:

$$\begin{pmatrix} A & B \\ C & D \end{pmatrix} = \begin{pmatrix} 1 & \mathrm{F2} \\ 0 & 1 \end{pmatrix} \cdot \begin{pmatrix} 1 & 0 \\ -1/\mathrm{F2} & 1 \end{pmatrix} \cdot \begin{pmatrix} 1 & d_6 \\ 0 & 1 \end{pmatrix} \cdot \begin{pmatrix} 1 & 0 \\ -1/\mathrm{F14} & 1 \end{pmatrix} \cdot \begin{pmatrix} 1 & d_5 \\ 0 & 1 \end{pmatrix}$$

$$\cdot \begin{pmatrix} 1 & 0 \\ -1/\mathrm{F13} & 1 \end{pmatrix} \cdot \begin{pmatrix} 1 & d_4 \\ 0 & 1 \end{pmatrix} \cdot \begin{pmatrix} 1 & 0 \\ -1/\mathrm{F12} & 1 \end{pmatrix} \cdot \begin{pmatrix} 1 & d_3 \\ 0 & 1 \end{pmatrix} \cdot \begin{pmatrix} 1 & 0 \\ -1/\mathrm{F11} & 1 \end{pmatrix}$$

$$\cdot \begin{pmatrix} 1 & d_2 \\ 0 & 1 \end{pmatrix} \cdot \begin{pmatrix} 1 & 0 \\ -1/\mathrm{F10} & 1 \end{pmatrix} \cdot \begin{pmatrix} 1 & d_1 \\ 0 & 1 \end{pmatrix} \cdot \begin{pmatrix} 1 & 0 \\ -1/\mathrm{F2} & 1 \end{pmatrix} \cdot \begin{pmatrix} 1 & \mathrm{F2} \\ 0 & 1 \end{pmatrix} \tag{20}$$

Solving the set of Supplementary equations (16-20), with regards of the provided information for focal lengths and distances between optical lenses (Supplementary Figure 2) results the final spot size to be r_2=1.82 µm and NA_f=0.148.

The ABCD law calculation is meant to be a free aberration investigation. Therefore, in order to optimize and reduce the aberration in the optical design provided in Supplementary Figure 2, we conducted a beam propagation simulation based on a commercially available code named Zemax and the result is provided in Supplementary Figure 3. We also note that all lenses that we used in the experiment were high precision aspheric lens and well aligned in the system.

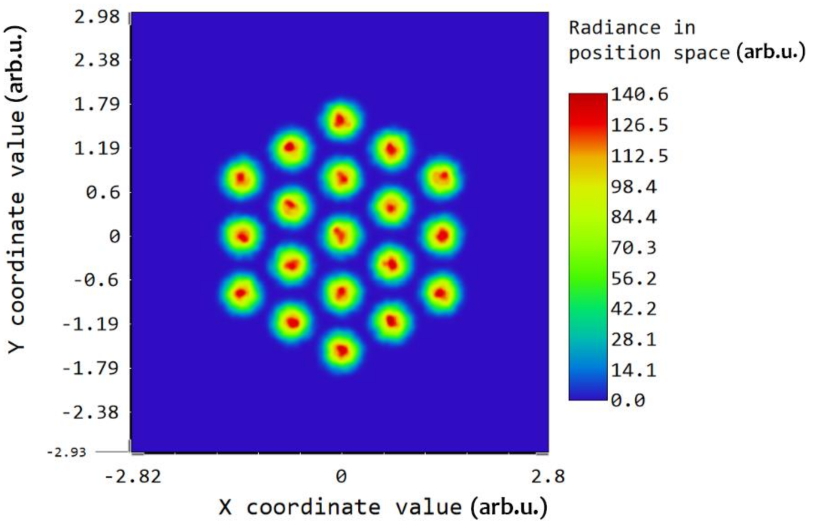

Supplementary Figure 3. Zemax simulation. The detector is the at the plane surface of the MCF. The optimization is done based on initial value for NA and spot size. The beams are well separated with the proper NA and spot size to re-inject back to the MCF.

Supplementary Note 5: Implementation of The Zeeman Terms Using a SLM

The Zeeman term is an external field factor in the Ising Hamiltonian. Initially the vertical polarized ML is injected into the SLs ($t < 0$). We then typically switch on the Zeeman terms together with the mutual couplings to let the CIM perform the calculation[3]. The Zeeman terms are implemented through modulation of the polarization state of the vertically polarized ML beam to let each core attain a small horizontal polarization component with different amplitudes. For this purpose, we used a liquid crystal reflective phase only SLM (HOLOEYE LC-R 720). In this configuration, we were able to program the amount of the horizontal polarization variation (or basically the χ_i factor) for different cores. The Zeeman terms thus can be calculated using Supplementary equation (8). The beam profile of the ML that was injected into the inner cladding of the MCF were assumed to be a Gaussian function. A geometrical fill factor together with a Gaussian beam-coupling factor was used to estimate the amount of the ML's power that was injected into each SL[13,14]. The SL powers were estimated from a technique based on integrated power measurement coupled with image processing using a MATLAB code. The offset angle was estimated after calibration of the LCOS-SLM that applies a polarization gradient across the vertically polarized input beam reflecting off of the SLM. The polarization state rotation of the ML after the SLM for the gray-levels from 0 to 255 were characterized using a rotation analyzer and a quarter-wave plate (QWP) as shown in Supplementary Figure 4. For Zeeman terms, blazed gratings were uploaded on the screen of the SLM as shown in Supplementary Figure 5.

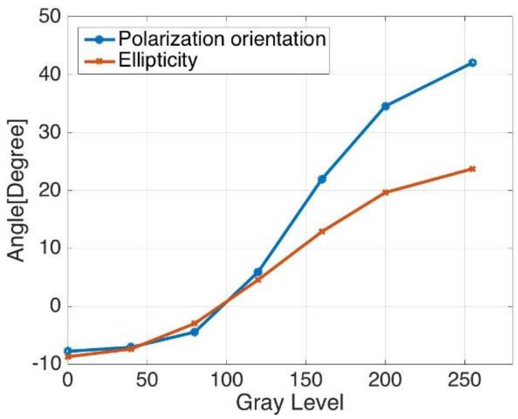

Supplementary Figure 4. Polarization orientation and ellipticity of the ML after the phase only reflective SLM set at blank screen with different gray-levels.

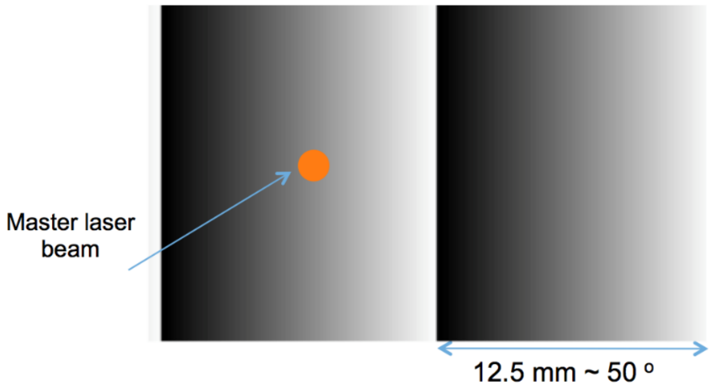

Supplementary Figure 5. Blazed grating uploaded to the SLM for the Zeeman terms in the systems and the spatial location of the on the SLM aperture. Polarization variation of ~2-3 º was encoded across the ML beam.

Considering the beam size of the ML to be ~1.5 mm and the corresponding polarization rotation for the gray-levels, we estimated a polarization rotation or deviation of up to ~3 º across the ML beam. We assume that the cores at the vertical polarized beam will be injected into the central SL and the cores on the left and right along a vertical line passing through the center of the MCF will experience up to ±1.5-degree polarization rotation. Using the estimated injected ML powers into each core together with SL powers of each core and the offset polarization angle we calculated the Zeeman terms.

Supplementary Note 6: Experimental Data for The Ising Hamiltonians

The Zeeman terms and mutual couplings were estimated based on the Supplementary equations (8) and (9) respectively at the time that they switched on. For the first experiment using a central symmetry coupling, instead of using the programmable SLMs to cross-couple the SL cores, we opted to use a lens and a retroreflector to establish the coupling. Ideally in this configuration, the front facet of the MCF will be imaged back to itself following inversion transformation. This will ensure that any SL will couple to the geometrically symmetric core in the 16-core array. Considering reflection and coupling losses, a maximum of 25 % of light would couple back to the MCF cores, which can disturb the states of the injection locked lasers. For that reason, we placed a variable optical attenuator (VOA) to couple a much smaller amount of the light back to the MCF cores. The optical phases among the SLs were considered as constant for the time being. For future work, we plan to measure and account for any phase differences that might be present. Although, we know the phase dependent parts of Supplementary equations (8) and (9) are positive since the difference phase between the ML and SLs is $-\boldsymbol{\pi}/2 \leq \varphi_i - \varphi_{\mathrm{M}} - \varphi_{\mathrm{M}i} \leq \boldsymbol{\pi}/2$ as well as the phase among SLs $-\boldsymbol{\pi}/2 \leq \varphi_i - \varphi_j - \varphi_{ji} \leq \boldsymbol{\pi}/2$ at the time the injection-locking is performed[4]. The optical power for each SL were estimated through a method based on the total power measurements of the MCF coupled with image processing techniques using a MATLAB script. The Gaussian beam profile is also assumed to estimate the coupling fill factor for the SL cores and the size of the imaged beam[13,14].

The Ising Hamiltonian that was obtained for the central symmetry couplings, where a flat mirror was used in order to implement the mutual couplings, is the following:

$$\lambda_{1\times 13} = \frac{1}{10^5}(56 \quad 13 \quad 76 \quad 63 \quad 11.2 \quad 49 \quad 36 \quad 67 \quad 24 \quad 65 \quad 9.1 \quad 11.3 \quad 74) \tag{21}$$

$$J_{13\times 13} = \frac{1}{100}\begin{pmatrix} 0 & 0 & 0 & 0 & 1.32 & 0 & 0 & 0 & 0 & 0 & 0 & 0 & 0 \\ 0 & 0 & 0 & 0 & 0 & 1.83 & 0 & 0 & 0 & 0 & 0 & 0 & 0 \\ 0 & 0 & 0 & 0 & 0 & 0 & 1.52 & 0 & 0 & 0 & 0 & 0 & 0 \\ 0 & 0 & 0 & 0 & 0 & 0 & 0 & 1.49 & 0 & 0 & 0 & 0 & 0 \\ 1.32 & 0 & 0 & 0 & 0 & 0 & 0 & 0 & 0 & 0 & 0 & 0 & 0 \\ 0 & 1.83 & 0 & 0 & 0 & 0 & 0 & 0 & 0 & 0 & 0 & 0 & 0 \\ 0 & 0 & 1.52 & 0 & 0 & 0 & 0 & 0 & 0 & 0 & 0 & 0 & 0 \\ 0 & 0 & 0 & 1.49 & 0 & 0 & 0 & 0 & 0 & 0 & 0 & 0 & 0 \\ 0 & 0 & 0 & 0 & 0 & 0 & 0 & 0 & 0 & 0 & 1.79 & 0 & 1 \\ 0 & 0 & 0 & 0 & 0 & 0 & 0 & 0 & 0 & 0 & 0 & 1.63 & 0 \\ 0 & 0 & 0 & 0 & 0 & 0 & 0 & 0 & 1.79 & 0 & 0 & 0 & 0 \\ 0 & 0 & 0 & 0 & 0 & 0 & 0 & 0 & 0 & 1.63 & 0 & 0 & 0 \\ 0 & 0 & 0 & 0 & 0 & 0 & 0 & 0 & 1 & 0 & 0 & 0 & 0 \end{pmatrix} \tag{22}$$

For the general couplings matrices, we have examined the Ising Hamiltonian with size of N=7, N=4 and N=3. Supplementary Figure 6 denotes the portions of the MCF that were used for the implementation of CIM with size of N=7, N=4 and N=3.

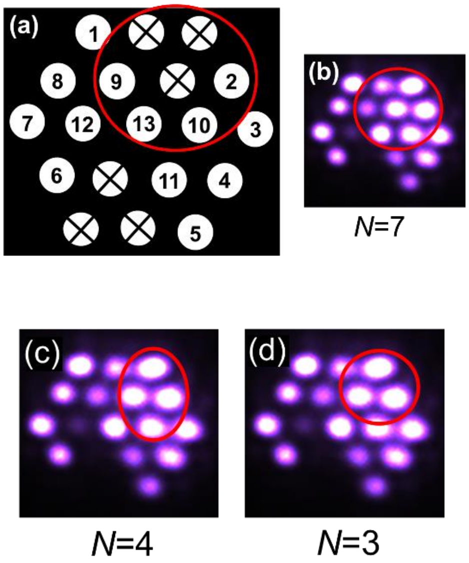

Supplementary Figure 6. The sections of the MCF that were used for the Ising Hamiltonian with size of N=7, N=4 and N=3.

The Ising Hamiltonian for the arbitrary connections using the SLM2 and SLM3 for the configuration provided in Fig. 7a is:

$$\lambda_{1\times 7} = \frac{1}{10^5}\begin{pmatrix}11 & 23 & 65 & 44 & 28 & 19 & 37\end{pmatrix} \tag{23}$$

$$J_{7\times 7} = \frac{1}{100}\begin{pmatrix} 0 & 1.5 & 0 & 0 & 0 & 0 & 1.18 \\ 1.5 & 0 & 1.02 & 0 & 0 & 0 & 0 \\ 0 & 1.02 & 0 & 1.19 & 0 & 0 & 0 \\ 0 & 0 & 1.19 & 0 & 0.89 & 0 & 0 \\ 0 & 0 & 0 & 0.89 & 0 & 1.31 & 0 \\ 0 & 0 & 0 & 0 & 1.31 & 0 & 0.91 \\ 1.18 & 0 & 0 & 0 & 0 & 0.91 & 0 \end{pmatrix} \tag{24}$$

The Ising Hamiltonian provided in Fig. 8a is the following:

$$\lambda_{1\times 3} = \frac{1}{10^5}\begin{pmatrix}23 & 83 & 7.2\end{pmatrix} \tag{25}$$

$$J_{3\times 3} = \frac{1}{100}\begin{pmatrix} 0 & 1.13 & 1.27 \\ 1.13 & 0 & 1.24 \\ 1.27 & 1.24 & 0 \end{pmatrix} \tag{26}$$

And for Fig. 9a:

$$\lambda_{1\times 4} = \frac{1}{10^5}\begin{pmatrix}9 & 12 & 67 & 29\end{pmatrix} \tag{27}$$

$$J_{4\times 4} = \frac{1}{100}\begin{pmatrix} 0 & 1.18 & 0 & 0.46 \\ 1.18 & 0 & 1.047 & 0 \\ 0 & 1.047 & 0 & 1.33 \\ 0.46 & 0 & 1.33 & 0 \end{pmatrix} \tag{28}$$

Supplementary Note 7: A Random Instance Ising Hamiltonian with Size of *N*=7

We performed another example for general coupling matrices with size of *N*=7. Supplementary Figure 7a shows the designed grating for this instance:

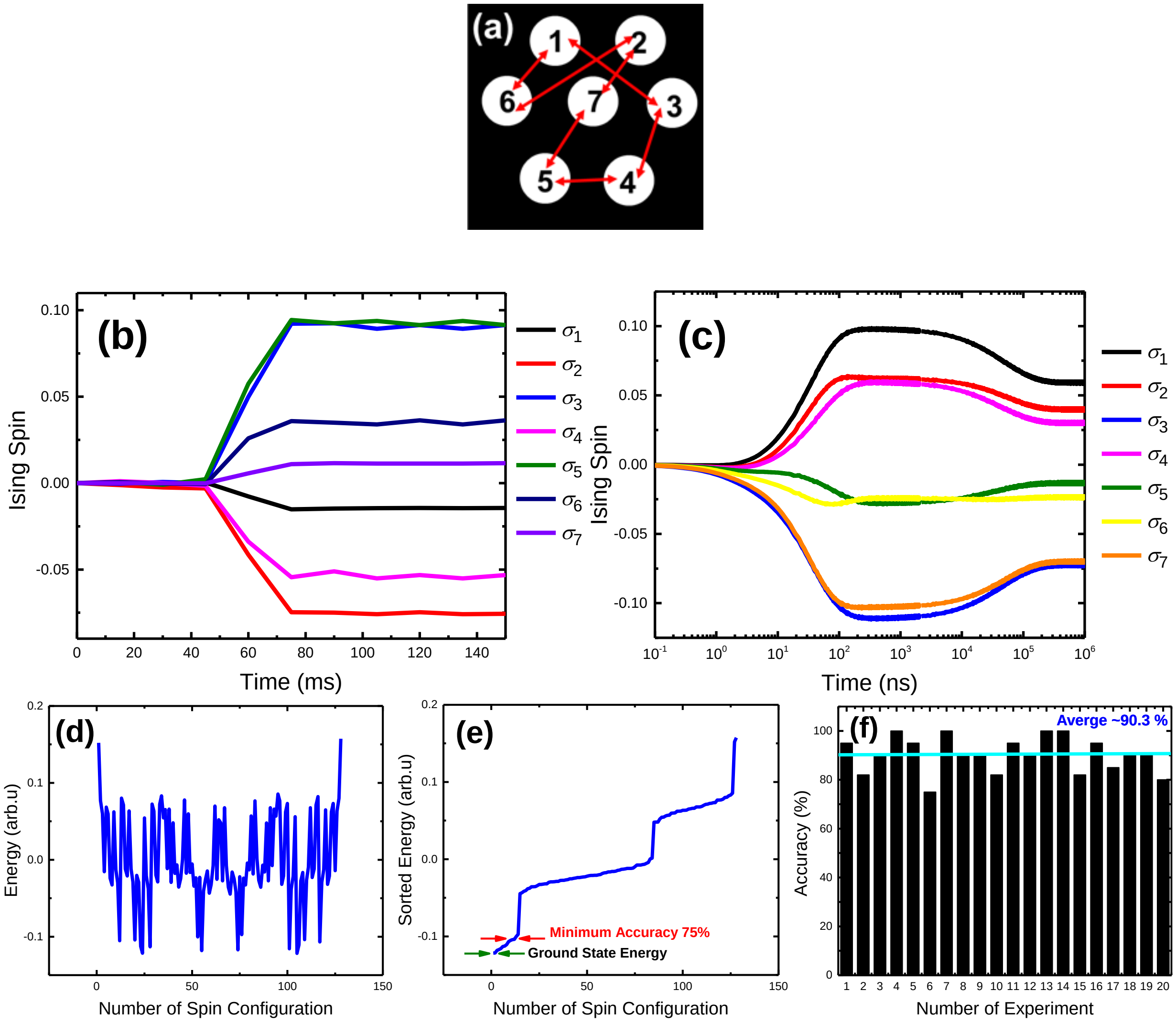

Supplementary Figure 7. Ising Hamiltonian with size of N=7. a The mutual coupling grating. **b** Experimental Ising spin evolution as the function of time. **c** Simulation and with a good agreement with Ising spin signs in Supplementary Figure 7b. **d** Ising energy landscape as the function of Ising spin configuration. **e** Sorted energy value versus all spin configurations (2^7). **f** Accuracy versus number of experiment. The overall average accuracy was 90.3%.

The Zeeman terms and mutual couplings were estimated to be:

$$\lambda_{1\times 7} = \frac{1}{10^5}\begin{pmatrix} 34 & 21 & 25 & 71 & 14 & 47 & 52 \end{pmatrix} \tag{29}$$

$$J_{7\times 7} = \frac{1}{100}\begin{pmatrix} 0 & 0 & 1.22 & 0 & 0 & 01.4 & 0 \\ 0 & 0 & 0 & 0 & 0 & 0.93 & 1.08 \\ 1.22 & 0 & 0 & 1.01 & 0 & 0 & 0 \\ 0 & 0 & 1.01 & 0 & 1.27 & 0 & 0 \\ 0 & 0 & 0 & 1.27 & 0 & 0 & 0.83 \\ 1.4 & 0.93 & 0 & 0 & 0 & 0 & 0 \\ 0 & 1.08 & 0 & 0 & 0.83 & 0 & 0 \end{pmatrix} \tag{30}$$

Supplementary Note 8: Scalability of the proposed CIM

In this section, we discuss the limitations of the proposed CIM scalability and possible solutions for them in order to increase the number of nodes and edges. As we noted in the manuscript the feed-back power among the SLs to implement the mutual couplings cannot be greater than an "specific percentage" of the SL's power. The stress on "specific percentage" is because this number can be arbitrary for different injection-locked laser systems, as long as the feed-back powers do not disturb the injection-locked status. Likewise, the feed-back power among the SLs cannot be lower than "specific percentage". This number indicates a minimum bound in order the CCD camera (measurement unit) can detect variation between n_R and n_L as the function of time. Let's consider a fully connected Ising Hamiltonian with size of N. The power of each SL (P) needs to be divided in fraction of $P/(N-1)$. Thus:

$$\alpha.P \leq \frac{\varepsilon.P}{N-1} \leq \beta.P \;\rightarrow\; \frac{\varepsilon}{\beta}+1 \leq N \leq \frac{\varepsilon}{\alpha}+1 \tag{31}$$

where ε denotes the diffraction efficiency of the SLMs. α and β (in percent) are the lower and upper bound fractions as described above, respectively. These numbers (α and β) are related to noise level of the SLs, ML and pump laser, sensitivity of the readout detectors and other macroscopic or microscopic imperfections. For instance, if the sensitivity of the readout detectors is high, α can be very small which results having ability to implement more number of nodes. If the injection-locking handling is maintained well such that the feed-back power to other SLs does not perturb the injection-locking status, we can in principle, program more Ising nodes. In the presented work, we found roughly $\alpha = 1\%$ and $\beta = 5\%$. This implies according to Supplementary equation (31), that we can program a fully connected Ising Hamiltonian with a maximum of N=101 nodes which implies the number of edges to be equal 5050 ($N_{edg} = \binom{N}{2} =$

$\frac{1}{2}N(N-1)$ and assuming ε =100%). SLMs with 94% efficiency are available. Using SLMs with 94% efficiency would allow us to increase the number of Ising nodes (N) to 95 for a fully connected Ising Hamiltonian supporting all the $N_{edg} = \binom{N}{2} = \frac{1}{2}N(N-1) = 4465$ edges. The SLMs that we used, had efficiency of ~ 2.13 % that results a fully connected Ising Hamiltonian with maximum size of N=3 which has been implemented and brought to the result section (Fig. 8).

Furthermore, using a better analog to digital converter (ADC) resolution for the CCD camera would result in detecting more accurately the ratio between n_R and n_L. Our current CCD camera (8 bits) can detect the change between n_R and n_L if the feed-back power ratios between SLs are greater than 1%. Using available CCD cameras with higher pixel depth (> 32 bits) would allow us to detect the change between n_R and n_L, for smaller than 1 % feed-back power ratios. Accordingly, the increase of the camera ADC resolution from 8 bits to higher bits (e.g., >32 bits), allows us to increase the number of a fully connected Ising nodes from N = 95 to higher nodes (e.g. N = 941 and with ~ 442,000 edges if hypothetically, the CCD camera can detect the change between n_R and n_L for the feed-back power ratios between SLs for greater than 0.1%). In both cases we assumed the SLMs have 94 % diffraction efficiency and node number estimations are based on Supplementary equation (31). Nevertheless, increasing the number of cores in the MCF is an essential task in order to scale the CIM to higher nodes (i.e., rectangular MCF with 12×12 = 144 and 6% wt Yb-doped phosphate glass fiber, manufactured by NP Photonics, Inc). Moreover, we should note that increasing the number of nodes in our proposed CIM, requires installing a more powerful pump power and ML. This dictates to engineer the cavity that can

handle heat generation in the MCF as well as maintaining the injection-locking status and other general challenges of cavity designing.

We would like also to mention that we have investigated further into what connectivity is needed in the Ising solver to encode (provably) hard instances of NP Hard problems. We have found that in an N-bit Ising problem (i.e., using N SLs in our system), of all the $N(N-1)/2 = O(N^2)$ possible connections, one only needs to have $O(N)$ connections present. This lets us scale to vastly larger Ising machines compared even to what we noted above in points A. and B. To be more specific, we looked at the problem of exact-cover-3 (EC3)[15,16], where one is tasked with assigning values (0 or 1) to N variables $x_1, \ldots, x_N$, such that M clauses -- each formed of three variables – have exactly one 1 and two 0s. An example N=10 instance could be the (M = 3) clauses: $\{(x_1, x_2, x_7,), (x_1, x_4, x_5,), (x_5, x_9, x_1)\}$, where one can easily eyeball a solution, $x_1 = 1$ and ($x_2 = \ldots = x_9 = x_{10} = 0$). This problem is NP Hard. Moreover, if an EC3 problem instance is picked at random for a large N, with $M = \mu N$ clauses, when $\mu \approx 0.6263$, that random problem instance is provably hard with probability approaching 1 as N tends to ∞. An EC3 problem instance can be mapped to minimizing an Ising function $E(s_1, s_2, \ldots, s_N) = \sum_{i=1}^{N} B_i S_i + \sum_{i \neq j}^{N} J_{ij} S_i S_j$ where B_i is the number of EC3 clauses bit i participates in, and J_{ij} is the number of EC3 clauses that bits i and j participate in together. In the above example, $B_1 = 3$, $B_5 = 2$ and $B_4 = 1$, $J_{1,2} = 1$, $J_{3,4}=0$, and so on. When a large EC3 problem instance is mapped to the Ising problem as above, the fraction of the connections that are non-zero is roughly $6\mu/N$, meaning the number of non-zero connections is roughly $6\mu/N \times [N(N-1)/2] = 3\mu(N-1) = O(N)$. So, encoding provably hard Ising problems drawn as above, will need $3\times0.6263 \times(N-1) \sim 1.88N$ of the $N(N-1)/2$ possible connections non-zero. For the N=1000 instance stated above, this means needing only 1880 edges in the J matrix non zero, as opposed to the 442,000 edges needed in a fully-connected Ising instance.

Supplementary References

1. Gillner, L., Bjork, G. & Yamamoto, Y. Quantum noise properties of an injection-locked laser oscillator with pump-noise suppression and squeezed injection. *Phys. Rev A* **41**(9), 5053–5065 (1990).

2. Haus, H. A. & Yamamoto, Y. Quantum noise of an injection-locked laser oscillator. *Phys. Rev. A* **29,** 1261–1274 (1984).

3. Utsunomiya, S., Takata, K. & Yamamoto, Y. Mapping of Ising models onto injection-locked laser systems. *Opt. Express* **19,** 18091–18108 (2011).

4. Takata, K., Utsunomiya, S. & Yamamoto, Y. Transient time of an Ising machine based on injection-locked lasers: contribution of locking bandwidth and Zeeman component. *New Journal of Physics* **14**, 013052 (2012).

5. Nguyen, D. T. et al. An Optical Ising Machine Based on Multi-core Fiber Lasers. *Proceeding IEE Summer Topical Conference* 11-13 (2016).

6. Li, L., Schülzgen, A., Li, H., Temyanko, V., Moloney, J. L., Peyghambarian, N. Phase-locked multicore all-fiber lasers: modeling and experimental investigation. *J. Opt. Soc. Am. B* **24,** 1721 (2007)

7. Suzuki, S., et.al. Image amplifier based on Yb3+-doped multi-core phosphate optical fiber. *Opt. Express* **15,** 3759-3765 (2007).

8. Anderson,J., et.al. Multi-core Fiber Lasers. *OSA Frontiers in Optics*, Paper LTu2H (2015).

9. Richardson, D.J., et al. Space division multiplexing in optical fibres. *Nat. Photon* **7,** 354 (2013).

10. Demir, V. et al. “Injection-Locked, single frequency, multi-core Yb-doped phosphate fiber laser,” *Sci. Rep* **9,** 356 (2019).

11. Kogelnik, H. &. Li, T. Laser beams and resonators. *Proceedings of the IEEE* **54,** Issue: 10, (1966).

12. Shaomin, W. Matrix methods in treating decentred optical systems. *Opt Quant Electron* 17: 1 https://doi.org/10.1007/BF00619988 (1985).

13. Savović, S. & Djordjevich, A. Method for calculating the coupling coefficient in step-index optical fibers. *Appl. Opt* **46,** 1477-1481 (2007).

14. Amara, M. K. & Melikechi, N. Coupling efficiency effects of launching a fringe pattern into a single-mode optical fiber. *J. Opt. Soc. Am. B* **20,** 2031-2036 (2003).

15. Garey., M. R. & Johnson., D.S. *Computers and Intractability: A Guide to the Theory of NP-Completeness*. (New York, NY, USA, W.H. Freeman, 1979)

16. Farhi, E. et al. A quantum adiabatic evolution algorithm applied to random instances of an NP-complete problem. *Science* **292,** 472–475 (2001).
